# Effect of an artificial intelligence-assisted tool on non-valvular atrial fibrillation anticoagulation management in primary care: protocol for a cluster randomized controlled trial

**DOI:** 10.1186/s13063-022-06250-8

**Published:** 2022-04-15

**Authors:** Xueying Ru, Lan Zhu, Yunhui Ma, Tianhao Wang, Zhigang Pan

**Affiliations:** 1 Department of General Practice, Zhongshan Hospital, Fudan University, Shanghai, China; 2 Xuhui District, Xietu Community Health Center, Shanghai, China

**Keywords:** Atrial fibrillation, Artificial intelligence, Clinical decision support system, Community health center, Guideline adherence, Cluster randomized controlled trial, Protocol

## Abstract

**Background:**

Atrial fibrillation (AF) is one of the most common cardiac arrhythmia diseases. Thromboembolic prophylaxis plays an essential role in AF therapy, but at present, general practitioners (GPs) are presumed to lack the knowledge and enthusiasm for AF management. Clinical decision support systems (CDSS), assisted by artificial intelligence, help primary care providers (PCPs) make quick, individualized, and correct clinical decisions. This primary aim of the study is to identify whether the promotion of the CDSS would improve the primary care provided to patients with AF. The secondary objectives are mainly to assess the health-economic and clinical benefits from using the CDSS, and the improvement of GPs’ AF management capability.

**Methods:**

This study will be a prospective cluster randomized controlled trial, conducted among 14 community health centers in Shanghai which were randomized as the intervention group and control group in a ratio of 1:1. The intervention group will use the CDSS in the consultation of patients with AF and the control group will maintain their usual care. The trial will include 498 patients with AF and the follow-up period will be 12 months. The primary outcome is set as the proportion of antithrombotic treatment prescriptions in agreement with recommendations in the latest China’s AF-related guidelines. The secondary outcomes are the frequency of consultation, the compliance rate of international normalized ratio (INR) in patients with warfarin, stroke morbidity, treatment compliance, medication satisfaction, and the cost-benefit analysis. Per-protocol (PP) analysis and the intention-to-treat (ITT) analysis will be conducted.

**Discussion:**

This study aims to identify whether the application of CDSS to manage patients with AF in China’s community health centers would bring benefits for patients, physicians, and health economics.

**Trial registration:**

Registry name: 非瓣膜性房颤社区AI辅助管理工具研发及推广效果研究 (Development and promotion of an AI-assisted tool for NVAF management in primary care); registry number: ChiCTR2100052307; registration date: Nov. 22^nd^, 2021; http://www.chictr.org.cn/showproj.aspx?proj=133849.

**Supplementary Information:**

The online version contains supplementary material available at 10.1186/s13063-022-06250-8.

## Background

### Introduction

Atrial fibrillation (AF) is one of the most common cardiac arrhythmia diseases [1], with more than 7.90 million patients with AF in China over 45 years of age, with a prevalence of 1.8% for general population and 3% for people over 75 years in 2020. In a rapidly aging population, it was estimated that the prevalence of AF would increase at least 2.5 times in the next 50 years [2] and the health risk to patients and the disease costs for the country would only increase as AF caused a twofold increase in all-cause mortality in women and a 1.5-fold increase in men [3]. Thromboembolic events, especially ischemic stroke, are the main issue [4], with the risk of ischemic stroke in patients with AF was four to five times higher than that in those without, and 70% of patients with a stroke caused by AF had poor outcomes [5]. Thromboembolic prophylaxis plays an essential role in AF management, with many studies indicating that both new oral anticoagulants (NOACs) and warfarin reduced all-cause mortality and the incidence of stroke and thromboembolic events among Asian and non-Asian patients [6]. However in 2013, the China Registry of Atrial Fibrillation (CRAF) study reported that among patients with AF in China with a high stroke risk where the CHA_2_DS_2_-VASc score exceeded two, only one in five received antithrombotic treatment, nearly 2/3 of patients received antiplatelet drugs, and almost one in 10 patients had no treatment at all [7]. The compliance rate of international normalized ratio (INR) in patients with warfarin was only 31.8%, based on patients with AF treated with warfarin whose time within therapeutic range (TTR) exceeded 60%.

Some scholars highlighted the view of “integrated care and stratified therapy,” which meant that patients could get access to comprehensive management in primary care, such as risk assessment, AF treatment, INR monitoring, health education, and treatment of comorbidity diseases, with upper hospitals responsible for the treatment of complications, emergencies, and operations [8]. China’s guidelines also suggested a comprehensive geriatric assessment (CGA) for old patients with AF, including fall risk, cognition, emotion, and psychology [9]. It is possible to move AF management work into community health centers, because of the progress China had made in primary care, the implementation of a two-way referral system [10], and the improvement in general practitioners’ (GPs’) work competence.

In daily practice, the process is not always followed, as GPs are busy at their work and they do not always get access to the latest guidelines promptly. Many GPs avoid AF management and some know little about the disease and antithrombotic therapies, so at present in China’s community health centers, AF is usually ignored by GPs, resulting in many patients seeking healthcare in superior hospitals or remaining in an unmanaged state [11].

Artificial intelligence (AI) collects massive amounts of medical data and knowledge, with technical advantages such as precise clustering and reinforcement learning and it can be advantageous to GPs by making data more accessible and freeing them from complex calculations [12]. Clinical decision support systems (CDSS), supported by computer algorithms, help primary care providers (PCPs) quickly make individualized and correct clinical decisions by combining patients’ data with guidelines and using evaluation tools in a short consultation [13]. The CDSS process can significantly improve GPs’ work efficiency and quality and reduce their workloads, making it a promising development in China’s primary care [14]. At present, CDSS are used mainly in the management of oncology and cardiovascular diseases [15–17], and AF antithrombotic management-associated CDSS are also be used in practice. Researches into the effect of CDSS on the management of AF have occurred in many developed countries, but due to the short follow-up time, inadequate sample sizes, and the imperfect design of their CDSS, many studies reported that CDSS improved the appropriate prescription of antithrombotic agents, and lowered the incidence of adverse events, but had no effect on the incidence of thromboembolism [18–23]. Unlike developed countries, the healthcare in developing countries is poor with a lower proportion of appropriate antithrombotic treatment in patients with AF [24] and initial consultation in primary care is not fully implemented. Therefore, it is necessary to study the effect of CDSS again in developing countries [25], exploring if any improvement would be obtained by applying AF management CDSS in these regions.

We cooperated with Ping An Healthcare and Technology Co., Ltd., referring to China’s latest AF guidelines, expert censuses, and suggestions, and combing some assessments of CGA [9], to develop an AI-assisted AF-related CDSS for GPs by integrating fusion data and knowledge modeling technology. In the previous pilot study, we applied the CDSS in one community health center as the software group, managing 53 patients for over 1 year and had a significantly increased proportion of appropriate anticoagulation compared with the control group which performed usual care. However, the pilot study included only two community health centers and a few patients. In this study, the CDSS was updated and optimized to solve the shortcomings discovered in the previous trial and more subjects will be enrolled from more community health centers.

### Objectives

The primary objective of this study is to identify whether the promotion of the CDSS would improve the primary care provided to patients with AF. The secondary objectives are mainly to assess the health-economic and clinical benefits from using the CDSS, and the improvement of GPs’ AF management capability. By promoting the CDSS, we hope to accelerate the process of prioritizing AF management in community health centers and the implementation of the two-way referral system.

## Methods

### Study design

This study is a prospective, paralleled, open-label, single-center cluster randomized controlled trial. The Ethics Committee of Zhongshan Hospital approved the present study protocol (Approval Number: B2021-579(2)). The study was registered in the Chinese Clinical Trial Registry, International clinical trials registry platform of the World Health Organization (Registration Number: ChiCTR2100052307). This protocol was designed and described according to the standard protocol item- Recommendations for Interventional Trials–Artificial Intelligence (SPIRIT-AI) extension published in 2019 [26] (Additional file 1) and the consolidated standards of reporting trials (CONSORT) 2010 statement: extension to cluster randomized trials [27].

### Patients and public involvement

Neither patients nor the public were or will be involved in the design, planning, conduct, or reporting of this study.

### Settings and participants

The requirements for using the CDSS in community health centers are that their servers of the hospital information systems (HISs) are at least with eight-core central processing unit, 32 GB RAM, hard drive of 500 G, and operating system of Centos 7.2. Fourteen community health centers willing to participant in this study in Baoshan District and Jing’an District of Shanghai were recruited by researchers. Baoshan District and Jing’an District are in the suburb and urban area of Shanghai, respectively, so are geographically representative (Additional file 2).

The participants are GPs and patients with AF in the selected community health centers. For GPs, the inclusion criteria are that they work in GP consulting rooms or the AF special consulting rooms in their community health center, are willing to participate in the study, and can complete the pre-training on AF management and CDSS use. They will be excluded if they rarely use computers in their daily work, or cannot complete the whole study.

Patients will be included if they are 18 years old or older, diagnosed with any type of AF or atrial flutter (ICD-10 codes shown in Additional file 3) during a consultation or in medical history, registered with GP for at least 1 year, and are willing to participate in the study and sign the informed consent (Additional file 4). They will be excluded if they are diagnosed with end-stage disease such as advanced tumors, or their expected survival time is less than 1 year, or are unable to cooperate with the study due to illiteracy or dementia, or cannot continually consult in the registered community health centers in the next 1 year due to objective conditions such as moving, or with valvular AF or prosthetic valves, or are participating in other clinical trials related to AF, or are pregnant or breastfeeding. Subjects can reserve the right to drop out due to any reason or without reason and at any time during the study. If any incident happens to the subjects, such as death, disability, or dementia, they could withdraw from the study after applying by their legal representatives and evaluating by researchers. Subjects who are pregnant during the follow-up and plan to give birth will be considered as withdrawal.

### Randomization and blinding

#### Sequence generation

Stratified randomization was conducted considering the location of each community health center.

#### Implementation

The researchers use a computer to generate two random sequences, allocated the eight community health centers in Baoshan District and six CHSs in Jing’an District, respectively, in a ratio of 1:1 into an intervention and a control group to receive CDSS intervention or usual care.

#### Allocation concealment mechanism

The allocation results of each community health center were told to the administrators by telephone. Each administrator only knows the result of his or her own site.

#### Blinding

The researchers, participants, and data analysts will not be blinded, but the participants will not be allowed to know the outcomes of this study.

### Interventions

#### Introduction

The intervention in this study is an AI-assisted CDSS on non-valvular AF management which was developed for this study in conjunction with Ping An Healthcare and Technology Co., Ltd. The CDSS was designed based on China’s latest AF treatment-related documents. It possesses the abilities to automatically evaluate the risk of stroke using CHADS_2_ score and CHADS_2_-VASc score and bleeding by HAS-BLED score, ORBIT score, and ATRIA score and provide initial treatment, follow-up, and dosage adjustment suggestions. It also contains the new functions of providing referral suggestions, anticoagulant instructions, and tips on the interaction between the selected anticoagulants and other drugs or food, assessing fall risk and balance using the Tinetti Scale and Self-rated Fall Risk Questionnaire (self-rated FRQ)), cognition by the Mini-Mental State Examination (MMSE), emotional and psychological disorders using the geriatrics depression scale-15 (GDS-15), and the confusion assessment method (CAM). It will only be used to provide evidence-based advice to physicians without any person-AI interaction and the final decisions will still be made by GPs. The Shenzhen Wang’an Computer Safety Checking & Measuring Technology Co., Ltd. examined the system security and the developers evaluated the stability and algorithm verification. The rationality of the decisions was checked by cardiological experts.

#### Preparation

Prior to be used, the CDSS will be embedded into the HIS of community health centers in the intervention group. The GPs will be trained in the use of the CDSS within the intervention group by developers from Ping An Healthcare and Technology Co.Ltd., and GPs of both groups will be trained in AF management by the researchers. After training, the training materials will be bound into a handbook and provided to the GPs for free.

#### Usage

After training, GPs in the intervention group can use the CDSS to manage patients with AF. It will capture the diagnosis code automatically after being linked with HIS, so when a patient is diagnosed as AF or atrial flutter, a pop-up reminder will show up. The interface of the CDSS will be presented after GPs click on the pop-up window. Referral indicators will be showed first to remind GPs of judging whether the patient needs to be referred to a superior hospital. The referral indications included immediate and general indicators [28]. The detail introductions are separated into initial and follow-up visit patients.

##### Initial patients

**Fig. 1:**
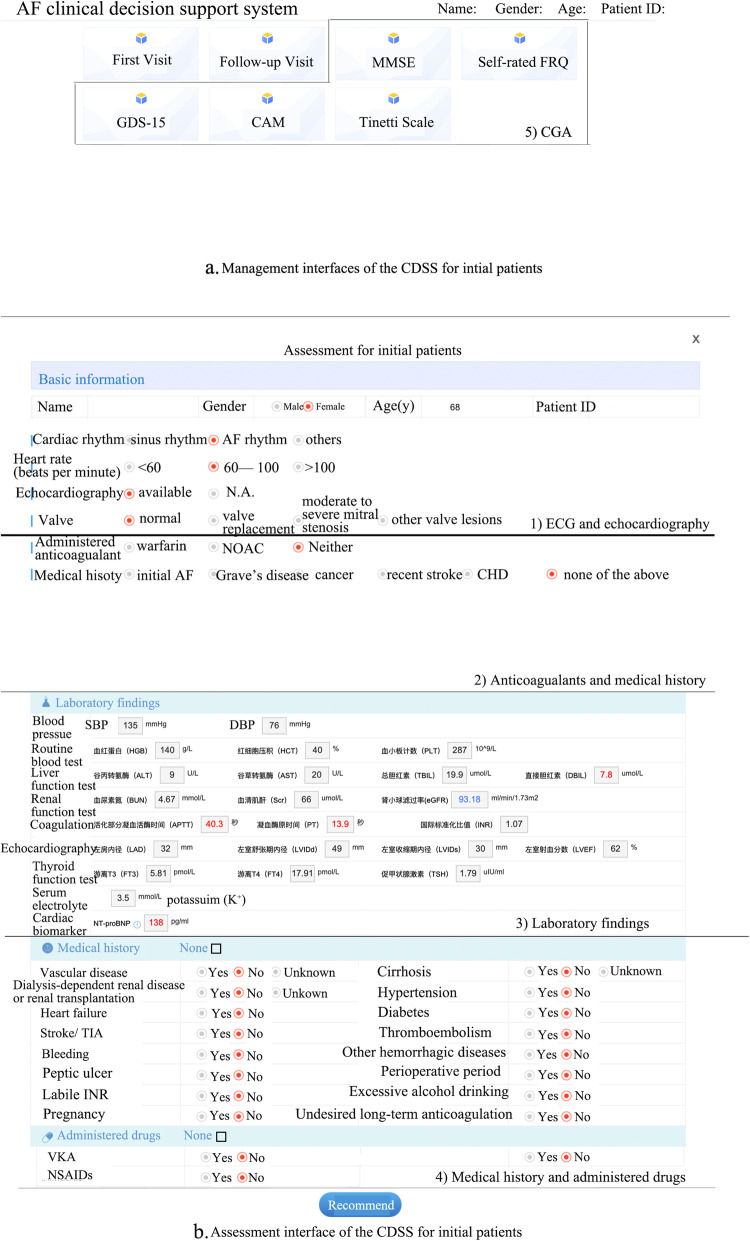
The interface of the CDSS for initial patients

**Fig. 2:**
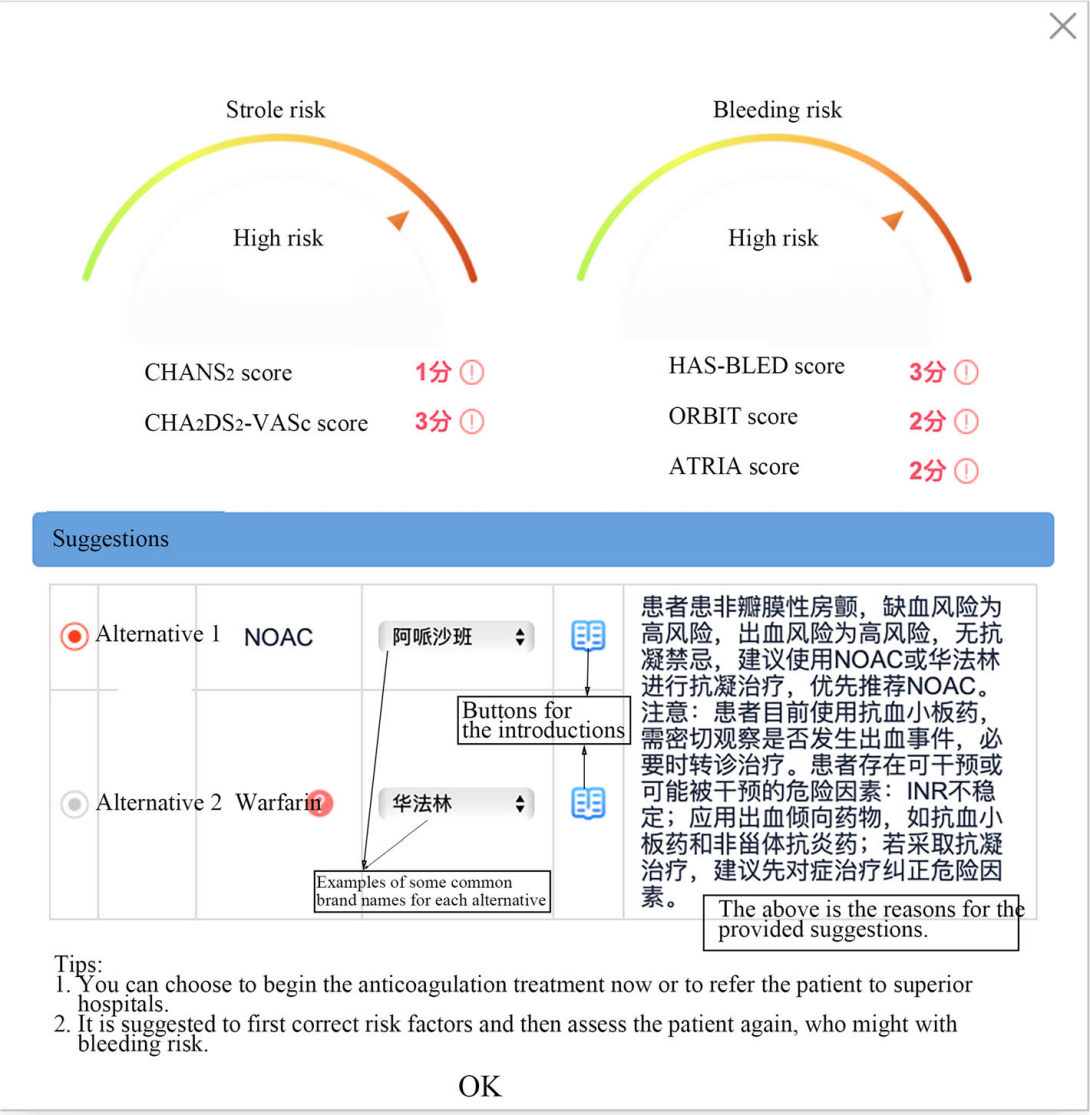
Example of suggestions for initial patients

Initial patients is defined as the patients who see the doctor in community health centers for the first time due to AF and have not been treated with any anticoagulants. The management interface is shown in Fig. 1a. The management procedures are in order:

1. Electrocardiogram (ECG) and echocardiography as in Fig. 1b. GPs will need to choose the appropriate option according to the patient’s ECG and echocardiography. If the ventricular rate is faster than 100 beats per minute or slower than 60 beats per minute, or if echocardiography is unavailable, or if a valvular disorder is suggested, the CDSS will advise referring the patients to superior hospitals to first regulate the ventricular rate, check the echocardiography, or treat the valvular disorders.
2. Those patients with ventricular rates ranging from 60 to 100 beats per minute and echocardiography reported as normal valvular function can continue with the CDSS. The anticoagulants, including warfarin and NOACs, and medical history including initial AF, Grave’s disease, cancer, recent stroke, and cardiovascular heart disease (CHD) are shown in Fig. 1b. If patients are taking such drugs, the CDSS will change to the follow-up interface. If the patients are diagnosed with any one of the diseases, the CDSS will advise referral.
3. Laboratory findings are shown in Fig. 1b. The CDSS will autoload relevant and latest laboratory findings within the last 3 months recorded in HIS. The required parameters are systolic and diastolic pressure in mmHg, hemoglobin (HGB) in g/L, hematocrit (HCT) as a percentage, platelet count (PLT) in units 10 [9]/L, alanine aminotransferase (ALT) and aspartate aminotransferase (AST) both in U/L, total bilirubin (TBIL) in μmol/L, direct bilirubin (DBIL) in μmol/L, blood urea nitrogen (BUN) in mmol/L, serum creatinine (Scr) in μmol/L, glomerular filtration rate (eGFR) as ml/min/1.73 m^2^), activated partial thromboplastin time (APTT) and prothrombin time (PT) both per second, left atrial diameter (LAD), left ventricular end-diastolic (LVIDd) and left ventricular end-systolic (LVIDs) all in mm, left ventricular ejection fraction (LVEF) as %, free T3 (FT3) and free T4 (FT4), both as pmol/L, thyroid-stimulating hormone (TSH) in IU/ml, serum potassium (K) as mol/L and NT-proBNP in pg/ml. Missing information in HIS will be corrected by GPs asking the patients.
4. Medical history and drugs are shown in Fig. 1b. Detailed medical history included the presence or otherwise of vascular disease, cirrhosis, dialysis-dependent renal disease or renal transplantation, hypertension, heart failure, diabetes, a major ischemic stroke within 2 weeks, bleeding, other hemorrhagic diseases, peptic ulcer, perioperative period, labile INR, excessive alcohol drinking, pregnancy, and undesired long-term anticoagulation. Current drugs include vitamin K antagonist (VKA), antiplatelet, and non-steroidal anti-inflammatory agents (NSAIDs).
5. CGA as seen in Fig. 1a, where if necessary, GPs can choose one or more assessments for patients, including fall risk and balance, cognition, and emotional and psychological disorders.
6. Finally, recommendations will be provided by the CDSS, including scores, risk levels, and suggestions on treatment, referral, and follow-up, according to the risk of stroke and bleeding (Fig. 2). If the assessment result of a patient is the need for anticoagulation, the CDSS will also provide the linkages of usage and cautions, instructions, and tips on the interaction between anticoagulants and other drugs or food to GPs (Fig. 2) [29].

##### Follow-up visit patients

**Fig. 3:**
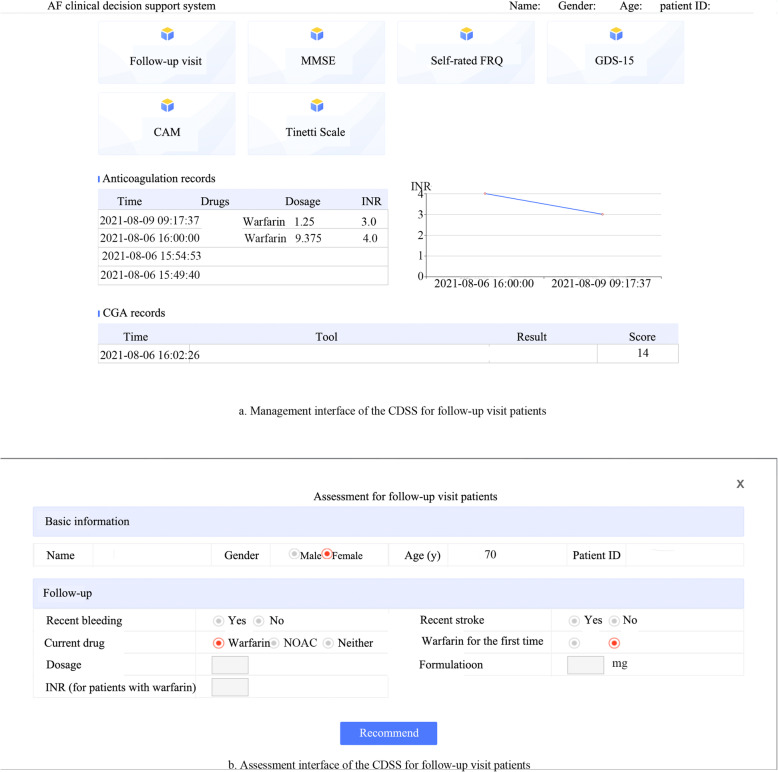
The interface of CDSS for follow-up visit patients

The follow-up patient management interface is shown in Fig. 3a and the example of result assessment is seen in Fig. 3b. For those with a recent stroke or bleeding, the CDSS will advise referring the patients and for those without, patients treated with NOAC or with no anticoagulants will be selected for continual follow-up and patients treated with warfarin will be provided recommendations based on their recent INR values.

### Outcomes

The primary outcome is the proportion of antithrombotic treatment prescriptions, in agreement with recommendations in the AF guidelines. It is defined as the agreement rate between the doctor’s anticoagulation prescriptions for newly diagnosed patients and the system’s recommendations, including prescribing anticoagulation drugs or recommending referral to superior hospitals for patients with anticoagulation indications, and refraining from anticoagulation for patients without such indications or with contraindications.

The safety outcomes, such as the incidence of bleeding events, all-cause mortality and in-patient events related to AF or AF complications will be reported. Referring to the Guideline of stroke prevention in Chinese patients with atrial fibrillation (2017), bleeding events are classified as minor and major bleeding [4].

The secondary outcomes contain seven items:

1. Frequency of consultation is defined as the average times of patients in each group seeking medical care due to AF in community health centers.
2. The INR compliance rate in patients with warfarin is defined as the number of patients with AF treated with warfarin whose TTR values exceed 65%, but excluding those whose INR values exceed 5.0 or are less than 1.5 at least twice in 6 months, or exceed 8.0 at least once [30]. For this calculation, INR values within the initial 6 weeks will be deleted and no less than 6-month INR values should be included.
3. Stroke morbidity during follow-up, including ischemic stroke and transient ischemic attack (TIA).
4. Medication satisfaction of patients, assessed with the Chinese version of the patient satisfaction questionnaire (PSQ-18) [31].
5. The GPs’ capability to manage AF, assessed using a self-administered questionnaire, “Knowledge-Attitude-Practice (KAP) questionnaire of community primary care physicians (PCPs) in anticoagulant therapy for non-valvular atrial fibrillation (NVAF) patients” [32], which was developed and validated by Delphi technique and the reliability and validity evaluation were confirmed by empirical research among GPs in Shanghai [33].
6. Cost-benefit analysis. The costs mainly include the time and labor costs caused by the design, installation, and maintenance of the CDSS compared with the control group, the excess time and labor costs led by GPs using the auxiliary tool and the loss of productivity due to patients’ longer visit time caused by using the CDSS.
7. The GPs’ acceptance of and rational and optimal advice on the CDSS. For this part, we plan to conduct semi-structured interviews

### Enrollment and follow-up

**Fig. 4:**
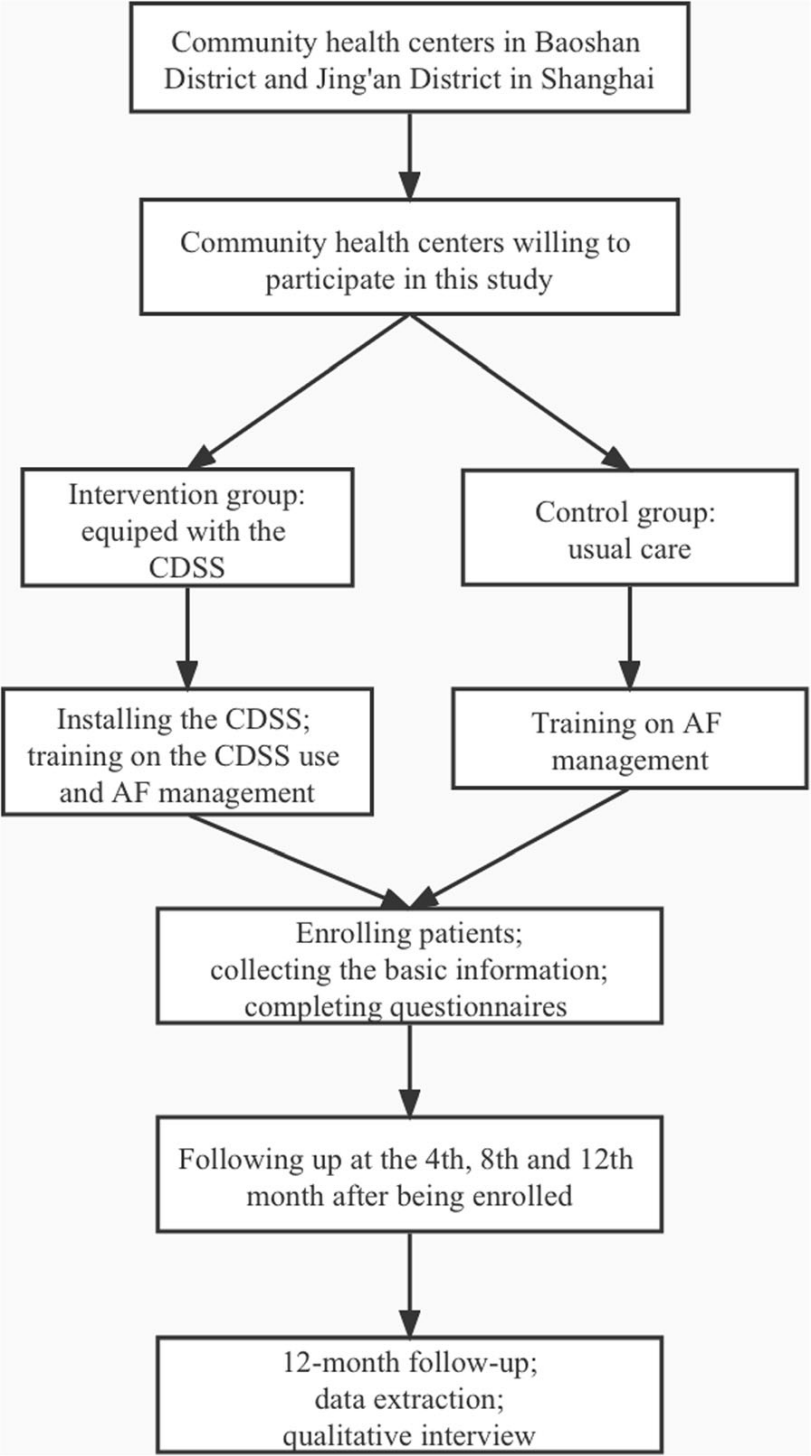
The study flowchart

GPs will be first enrolled, trained, and invited to complete the same KAP questionnaire on antithrombotic treatment three times before and after the training and at the end of the study. In the last month, one GP in each community health center of the intervention group will be randomly selected for a semi-structured interview. After training, GPs can enroll patients with AF that meet all the inclusion criteria and none of the exclusion criteria into this study, after signing the informed content. The follow-up will last for 12 months. Within the first week and the fourth, eighth, and twelfth months after enrolling, patients will be required to finish the PSQ-18 questionnaires. Patients will be contacted by telephone at the last week of a month or the first week of the next month, if they do not revisit their doctors in that month. One free INR point-of-care testing (POCT) collecting finger-stick blood will be provided to patients who complete all the 1 year time’s follow-up and the reasons for patients not finishing the study will be collected if possible. The study flowchart is shown in Fig. 4.

### Auditing

At each follow-up, the procedures will be checked in the 14 research settings and subjects who return uncompleted questionnaires will be invited to complete them.

### Data collection and management

Patients’ basic information will be collected by GPs with a questionnaire. Patients’ information during follow-up will be accessible in the health records and supplements by questionnaires if necessary. The data from the questionnaires will be generated in each follow-up, the follow-up date will be indicated, and the questionnaires will be signed by the investigators and double-entered into the Epidata3.0 database by two researchers. The interview content from GPs will be recorded by audio and verbally at the same time after getting informed consent. Two researchers will sum up the conversation and extract the key information related to the study aim independently. Disagreements will be resolved through discussion or by a third person. This study has no composition of data monitoring committee (DMC) because other data will be reserved in the information security department as parts of patients’ medical records. After completing the follow-up, we will apply to the Network Information Security Center of Baoshan District and Jing’an District Health Commission. The codes of the CDSS will not be accessible at any time except installation and necessary maintenance. When installing the CDSS, developers will cooperate with the programmers of HIS providers and minimize the risk of data leakage. If there are any bugs or data leakage, all people will stop using the CDSS until the problem is solved and the ethics committee approves resumption. The GPs can contact the developers and researchers at any time if they encounter any difficulties and a response will be given within 24 h. For severe adverse events, researchers will report them to the ethics committee within 24 h. If it is attributed to the CDSS, the ethics committee, and the principal investigator (PI) can terminate prematurely and conduct interim analyses. And we will compensate those who suffer harm in accordance with the Chinese law, if necessary.

### Sample size

A previous study reported that 12.64% of patients with AF received appropriate anticoagulants in community health centers of Shanghai [34]. Intracluster correlation coefficient (ICC) was 0.02 [35], the power (1-β) was 90%, and the double-sided significance (α) was 0.05. In the pilot study, the proportion of appropriate anticoagulation increased to 28% in the intervention group, so we expected the proportion to be 28% in this study. Seven community health centers are in each group and we plan to enroll 30 patients from each community health center in the control group, meaning a sample size of 448, or 498 to allow for a 10% loss to follow-up.

### Statistical analysis

Categorical data will be reported as frequencies and percentages (*n*, %), and a chi-square test will be used to detect any significant differences. Continuous data will be reported as mean ± SD (standard deviation) and compared using a *t*-test if it is on the Gaussian distribution. Otherwise, the data will be reported as the median (IQR (interquartile range)) and compared using the Mann-Whitney *U* test. Linear regression model will be used to compare the continuous data between the distinct groups, considering the interaction of time and allocation. Logistic regression model will be used to detect the effect of the interventions on the primary outcome. Kaplan-Meier (Log-rank test) and Cox proportional hazard models are to be used for the secondary outcomes and safety outcomes. To simplify the analysis, the follow-up time will be recorded as a unit of a month and regardless of start or end. The time will be calculated from this month, if it occurs in the former month; otherwise, it will be calculated from the next month. All outcomes will be evaluated with Mantel-Haenszel statistics and adjusted for the effect of clustering.

Per-protocol (PP) analysis and the intention-to-treat (ITT) analysis will be conducted after filling missing values by multiple imputations. The subgroup analyses will be conducted by whether the CDSS is installed and used in all general clinics or only the AF special consulting room. The sensitivity analyses will be performed by age strata, gender, resident district, and anticoagulants used.

All statistical analyses will be performed with IBM SPSS Statistics, version 26.0 (SPSS Inc) and R (version 4.0.5). A two-tailed *P* < 0.05 will be considered statistically significant.

## Discussion

### The necessity to conduct the study

High fatality and disability always result in heavy medical burden. A systematic review published in 2011 indicated that the medical expense caused by AF management was 10,100 to 14,200 dollars per person in the USA and 450 to 3000 euros per person in the EU [36]. In China, according to the data in 2012, it was estimated that the annual treatment cost of AF cerebral ischemia was 4.9 billion yuan, accounting for 10.6% of the total economic cost of strokes [37]. This was closely associated with the low proportion of appropriate antithrombotic treatment prescriptions. In the future China’s medical model blueprint, most patients with AF will get access to antithrombosis medical care in primary care, but according to previous studies, only 4.24 to 12.64% of patients with AF received any kind of care in community health centers [38, 39] due to GPs’ concern about bleeding and reluctance to promote awareness of thromboembolic prophylaxis [4, 33]. To implement “integrated care and stratified therapy,” the first step is to strengthen GPs’ AF management capability.

Xietu Road Community HealthThe developing trend of AF forced the development of smart models for diagnosis and treatment using AI [40, 41]. Some related studies suggested that although using CDSS failed to meet the expected effects, such as remarkably improving the clinical outcomes and increasing the proportion of oral anticoagulant (OAC) use, most GPs accepted such assisted tools well [42, 43]. In the previous pilot study conducted by us, the proportion of patients in the intervention group treated following the recommendations in the AF guidelines was higher than that in the control group. The GP in the intervention group of the pilot study thought that using the CDSS strengthened her confidence to prescribe necessary anticoagulants to patients, and it was also useful when she taught patients on the precautions of anticoagulation. Thus, we predicted that using CDSS in China’s primary care would benefit both patients with AF and GPs.

### Innovation of this study

As both GPs and patients with AF are considered as the subjects in this study, it had two differences from prior studies. First, some assessments of CGA is added based on the experience from the pilot study and China’s expert consensus on the management of atrial fibrillation in elderly population (2016), including the assessment of falls. Holt et al. reported that their study of “automated risk assessment for stroke in atrial fibrillation (AURAS-AF)” failed to increase the prescription of OAC because the CDSS used did not overcome the GP’s fears of bleeding events resulting from frailty and fall risk of their patients. Thus, the CDSS used in this study was updated accordingly. Second, it could be integrated into HIS, automatically identify pre-set keywords and diagnosis codes, and get immediate access to the individual’s related medical records. These functions are advanced in China and other developing countries. The study is designed scientifically and appropriately with representative subjects, and the outcomes involved GPs, patients, and health economics.

### Expectation of results

For GPs, the promotion of CDSS is expected to help assess patients with AF, make reasonable decisions quickly and accurately, and improve their AF management greatly. For patients with AF, it will make medical care more convenient and confer clinical benefits. The CDSS will also increase the proportion of patients accepting AF management in primary care and accelerate the process of assigning the AF management work to community health centers, as well as implementing the two-way referral system.

## Conclusion

The CDSS we developed aims to help GPs in primary care manage patients with AF better, which possesses the abilities to automatically evaluate the risk of stroke and bleeding and provide treatment suggestions. We plan to conduct a cluster randomized controlled trial, exploring whether using CDSS in China’s primary care can improve the capability and enthusiasm of GPs to manage AF regularly, and increase clinical benefits for patients with AF.

## Trial status

**Fig. 5:**
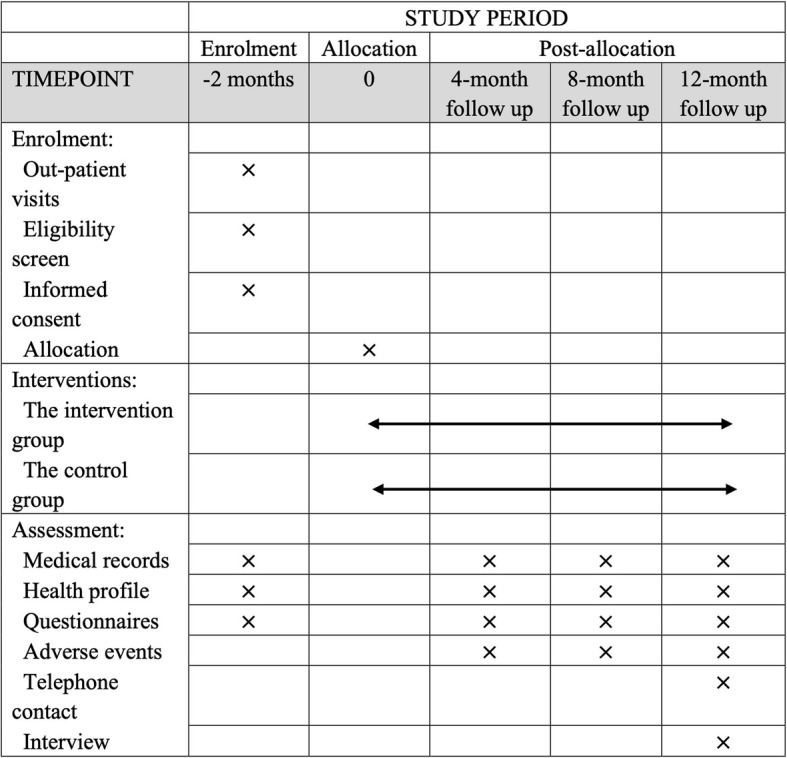
Schedule of enrollment, interventions, and assessments according to the SPIRIT 2013 Statement: Defining Standard

This publication is based on version 1.2 of the trial protocol dated Oct. 15, 2021. It is planned to enroll participants in Jan. 2022, and to follow up until Jan. 2023 (Fig. 5).

## Supplementary Information

**Additional file 1.** SPIRIT-Al checklist.

**Additional file 2.** Community health centers participating in the study.

**Additional file 3.** ICD-10 codes for AF and atrial flutter.

**Additional file 4.** Informed consent.

**Additional file 5.** Ethical approval document.

**Additional file 6.** Copy of the original funding documentation.

## Data Availability

No additional data are available.

## Abbreviations

AF: Atrial fibrillation
AI: Artificial intelligence
ALT: Alanine aminotransferase
APTT: Activated partial thromboplastin time
AST: Aspartate aminotransferase
BUN: Blood urea nitrogen
CAM: Confusion Assessment Method
CDSS: Clinical decision support systems
CGA: Comprehensive geriatric assessment
CHD: Cardiovascular heart disease
CI: Confidence interval
CRAF: China Registry of Atrial Fibrillation
DBIL: Direct bilirubin
DMC: Data monitoring committee
ECG: Electrocardiogram
eGFR: Glomerular filtration rate
FT3: Free T3
FT4: Free T4
GDS-15: Geriatrics Depression Scale-15
GP: General practitioner
HCT: Hematocrit
HGB: Hemoglobin
HIS: Hospital information system
ICC: Intracluster correlation coefficient
ICD-10: International Classification of diseases
INR: International normalized ratio
IQR: Interquartile range
ITT: Intention-to-treat
KAP: Knowledge-Attitude-Practice
LAD: Left atrial diameter
LVEF: Left ventricular ejection fraction; left ventricular end-diastolic
LVIDs: Left ventricular end-systolic
MMSE: Mini-Mental State Examination
NOAC: New oral anticoagulants
NSAIDs: Antiplatelet and non-steroidal anti-inflammatory agents
NVAF: Non-valvular atrial fibrillation
OAC: Oral anticoagulants
PCP: Primary care provider
PI: Principal investigator
PLT: Platelet count
POCT: Point-of-care testing
PP: Per-protocol
PSQ-18: Patient Satisfaction Questionnaire
PT: Prothrombin time
Scr: Serum creatinine
SD: Standard deviation
SPIRIT-AI: Standard Protocol Items: Recommendations for Interventional Trials–Artificial Intelligence
TIA: Transient ischemic attack
TBIL: Total bilirubin
TSH: Thyroid-stimulating hormone
TTR: Time within therapeutic range
VKA: Vitamin K antagonist

## References

[1] Benjamin EJ, Muntner P, Alonso A, Bittencourt MS, Callaway CW, Carson AP (2019). Heart Disease and Stroke Statistics-2019 Update: A Report From the American Heart Association. Circulation..

[2] Zhou Z, Hu D, Chen J, Zhang R, Li K, Zhao X (2004). An epidemiological survey of atrial fibrillation in China. Chin J Int Med.

[3] Chiang CE, Okumura K, Zhang S, Chao TF, Siu CW, Wei Lim T (2017). 2017 consensus of the Asia Pacific Heart Rhythm Society on stroke prevention in atrial fibrillation. J Arrhythm..

[4] Shu Z, Minyan Y, Congxin H, Dejia H, Kejiang C, Jun Z (2018). Guideline of stroke prevention in Chinese patients with atrial fibrillation (2017). Chin J Cardiac Arrhyth..

[5] Wang Z, Chen Z, Wang X, Zhang L, Li S, Tian Y (2018). The Disease Burden of Atrial Fibrillation in China from a National Cross-sectional Survey. Am J Cardiol..

[6] Wang KL, Lip GY, Lin SJ, Chiang CE (2015). Non-Vitamin K Antagonist Oral Anticoagulants for Stroke Prevention in Asian Patients With Nonvalvular Atrial Fibrillation: Meta-Analysis. Stroke..

[7] Committee of Cardiocerebral and Vascular Disease of Chinese Gerontological Society Chinese Expert Consensus Group on Application of New Oral Anticoagulation Drugs in Patients with Nonvalvular Atrial Fibrillation (2014). The current situation and future of the atrial fibrillation anticoagulation in China. Chin J Cardiovasc Res.

[8] Kirchhof P (2017). The future of atrial fibrillation management: integrated care and stratified therapy. Lancet..

[9] Population WCfECotMoafiE, Society CG, Geriatrics EBoCJo (2016). Expert consensus on the management of atrial fibrillation in elderly population(2016). Chinese. J Geriatr..

[10] National Health Commission of the People’s Republic of China, National Administration of Traditional Chinese Medicine. Notice on Printing and Distributing the Technical Plan for Hierarchical Diagnosis and Treatment of Atrial Fibrillation. http://www.nhc.gov.cn/yzygj/s3594q/201909/592e8b8ac8044617bc9ed9186c2ee19f.s.html. Accessed 10 Sept 2019.

[11] Gu J, Song H, Zhou Y, Cheng W, Dai L, Li Z (2013). Management window for patients with atrial fibrillation in community health center. Chin J Gen Practitioners..

[12] He K, Wu Z (2020). Advances in artificial intelligence in prediction of atrial fibrillation. Chin J Clin Thorac Cardiovasc Surg.

[13] Kawamoto K, Houlihan CA, Balas EA, Lobach DF (2005). Improving clinical practice using clinical decision support systems: a systematic review of trials to identify features critical to success. Bmj..

[14] Wess ML, Saleem JJ, Tsevat J, Luckhaupt SE, Johnston JA, Wise RE (2011). Usability of an atrial fibrillation anticoagulation decision-support tool. J Prim Care Community Health..

[15] Bright TJ, Wong A, Dhurjati R, Bristow E, Bastian L, Coeytaux RR (2012). Effect of clinical decision-support systems: a systematic review. Ann Intern Med..

[16] Kessler ME, Carter RE, Cook DA, Kor DJ, McKie PM, Pencille LJ (2017). Impact of electronic clinical decision support on adherence to guideline-recommended treatment for hyperlipidaemia, atrial fibrillation and heart failure: protocol for a cluster randomised trial. BMJ Open..

[17] Klarenbeek SE, Weekenstroo HHA, Sedelaar JPM, Fütterer JJ, Prokop M, Tummers M (2020). The Effect of Higher Level Computerized Clinical Decision Support Systems on Oncology Care: A Systematic Review. Cancers (Basel).

[18] Arts DL, Abu-Hanna A, Medlock SK, van Weert HC (2017). Effectiveness and usage of a decision support system to improve stroke prevention in general practice: A cluster randomized controlled trial. PLoS One..

[19] Cox JL, Parkash R, Foster GA, Xie F, MacKillop JH, Ciaccia A (2020). Integrated Management Program Advancing Community Treatment of Atrial Fibrillation (IMPACT-AF): A cluster randomized trial of a computerized clinical decision support tool. Am Heart J..

[20] Eckman MH, Lip GY, Wise RE, Speer B, Sullivan M, Walker N (2016). Impact of an Atrial Fibrillation Decision Support Tool on thromboprophylaxis for atrial fibrillation. Am Heart J..

[21] Karlsson LO, Nilsson S, Bång M, Nilsson L, Charitakis E, Janzon M (2018). A clinical decision support tool for improving adherence to guidelines on anticoagulant therapy in patients with atrial fibrillation at risk of stroke: A cluster-randomized trial in a Swedish primary care setting (the CDS-AF study). PLoS Med..

[22] Piazza G, Hurwitz S, Galvin CE, Harrigan L, Baklla S, Hohlfelder B (2020). Alert-based computerized decision support for high-risk hospitalized patients with atrial fibrillation not prescribed anticoagulation: a randomized, controlled trial (AF-ALERT). Eur Heart J..

[23] van Doorn S, Rutten FH, O'Flynn CM, Oudega R, Hoes AW, Moons KGM (2018). Effectiveness of CHA(2)DS(2)-VASc based decision support on stroke prevention in atrial fibrillation: A cluster randomised trial in general practice. Int J Cardiol..

[24] Steinberg BA, Gao H, Shrader P, Pieper K, Thomas L, Camm AJ (2017). International trends in clinical characteristics and oral anticoagulation treatment for patients with atrial fibrillation: Results from the GARFIELD-AF, ORBIT-AF I, and ORBIT-AF II registries. Am Heart J..

[25] Guo Y, Lane DA, Wang L, Zhang H, Wang H, Zhang W (2020). Mobile Health Technology to Improve Care for Patients With Atrial Fibrillation. J Am Coll Cardiol..

[26] Cruz Rivera S, Liu X, Chan AW, Denniston AK, Calvert MJ, Spirit AI (2020). Guidelines for clinical trial protocols for interventions involving artificial intelligence: the SPIRIT-AI extension. Nat Med..

[27] Eldridge SM, Chan CL, Campbell MJ, Bond CM, Hopewell S, Thabane L (2016). CONSORT 2010 statement: extension to randomised pilot and feasibility trials. BMJ..

[28] Chinese Medical Association, Chinese Medical Journals Publishing House, Chinese Society of General Practice, Editorial Board of Chinese Journal of General Practitioners of Chinese Medical Association, Disease EGoGfPCoC (2020). Guideline for primary care of atrial fibrillation (2019). Chin J Gen Pract.

[29] Chinese Cardiovascular Disease Society Editorial Board Committee of Cardiocerebral and Vascular Disease of Chinese Gerontological Society (2013). Chinese Expert Consensus on the Clinical Application of Warfarin (Basic Version). Chin J Gen Practitioners.

[30] Gallagher AM, Setakis E, Plumb JM, Clemens A, Staa TV (2011). Risks of stroke and mortality associated with suboptimal anticoagulation in atrial fibrillation patients. Thromb Haemost..

[31] Wu S (2016). A Study of Patients’ Satisfaction between 3-A-Grade General Hospital and Community Health Service Centre in Guangzhou.

[32] Ye S, Pan Z, Liu W, Zhu L (2018). General Practitioners’ KAP Questionnaire on Anticoagulation in Patients with Nonvalvular Atrial Fibrillation Using Delphi Method. Chin Gen Pract.

[33] Ye S, Wang T, Liu A, Yu Y, Pan Z, Gu J (2020). A study of knowledge, attitudes, and practices of primary care physicians toward anticoagulant therapy in patients with non-valvular atrial fibrillation in Shanghai, China. BMC Fam Pract..

[34] Guo Y (2020). Analysis of current status of anticoagulant therapy in patients with atrial fibrillation in community. Diet health..

[35] Guo Y, Lane DA, Wang L, Chen Y, Lip GYH (2019). Mobile Health (mHealth) technology for improved screening, patient involvement and optimising integrated care in atrial fibrillation: The mAFA (mAF-App) II randomised trial. Int J Clin Pract..

[36] Wolowacz SE, Samuel M, Brennan VK, Jasso-Mosqueda JG, Van Gelder IC (2011). The cost of illness of atrial fibrillation: a systematic review of the recent literature. Europace..

[37] Zhang L, Yin C, Hu S (2013). The Illness Burden Brought by Atrial Fibrillation in China. Chin Health Econ.

[38] Qi Y, Jin X, Li S, Cao J, Chen Y, Tang B (2018). Epidemiological characteristics and anticoagulant therapy status of atrial fibrillation in elderly community residents in Shanghai. Chin J Clin Med.

[39] Xue H, Zeng G, Yingmin L (2020). Survey on the treatment of anticoagulation in 212 patients with atrial fibrillation in the community of Shanghai. South China J Cardiovasc Dis.

[40] Kornej J, Börschel CS, Benjamin EJ, Schnabel RB (2020). Epidemiology of Atrial Fibrillation in the 21st Century: Novel Methods and New Insights. Circ Res..

[41] Wang QC, Wang ZY (2020). Big Data and Atrial Fibrillation: Current Understanding and New Opportunities. J Cardiovasc Transl Res..

[42] Holt TA, Dalton AR, Kirkpatrick S, Hislop J, Marshall T, Fay M (2018). Barriers to a software reminder system for risk assessment of stroke in atrial fibrillation: a process evaluation of a cluster randomised trial in general practice. Br J Gen Pract..

[43] Wang Y, Bajorek B (2018). Selecting antithrombotic therapy for stroke prevention in atrial fibrillation: Health professionals’ feedback on a decision support tool. Health Informatics J..

