# Supplementary material for: Effect of an artificial intelligence-assisted tool on non-valvular atrial fibrillation anticoagulation management in primary care: protocol for a cluster randomized controlled trial
Source: Trials. 2022 Apr 15;23:316. doi: 10.1186/s13063-022-06250-8 (PMC9013112; doi:10.1186/s13063-022-06250-8)
Supplement: Supplementary file 1 — Additional file 1. SPIRIT-Al checklist. [file 13063_2022_6250_MOESM1_ESM.pdf]

Table 1 | SPIRIT-AI checklist

Section	Item	SPIRIT 2013 item[a]	SPIRIT-AI item		Addressed on page number[b]
Administrative information					
**Title**	1	Descriptive title identifying the study design, population, interventions, and, if applicable, trial acronym	SPIRIT-AI 1 (i) Elaboration	Indicate that the intervention involves artificial intelligence/machine learning and specify the type of model.	Page 1
			SPIRIT-AI 1 (ii) Elaboration	Specify the intended use of the AI intervention.	Page 1
**Trial registration**	2a	Trial identifier and registry name. If not yet registered, name of intended registry			Page 4&8
	2b	All items from the World Health Organization Trial Registration Dataset			Throughout the paper, pages 1-35
**Protocol version**	3	Date and version identifier			Page 24
**Funding**	4	Sources and types of financial, material, and other support			Page 27
**Roles and responsibilities**	5a	Names, affiliations, and roles of protocol contributors			Page 1,2&27
	5b	Name and contact information for the trial sponsor			Page 2
	5c	Role of study sponsor and funders, if any, in study design; collection, management, analysis, and interpretation of data; writing of the report; and the decision to submit the report for publication, including whether they will have ultimate authority over any of these activities			Page 27
	5d	Composition, roles, and responsibilities of the coordinating center, steering committee, endpoint adjudication committee, data management team, and other individuals or groups overseeing the trial, if applicable (see Item 21a for data monitoring committee)			Page 18
Introduction					
**Background and rationale**	6a	Description of research question and justification for undertaking the trial, including summary of relevant studies (published and unpublished) examining benefits and harms for each intervention	SPIRIT-AI 6a (i) Extension	Explain the intended use of the AI intervention in the context of the clinical pathway, including its purpose and its intended users (for example, healthcare professionals, patients, public).	Page 6-8
			SPIRIT-AI 6a (ii) Extension	Describe any pre-existing evidence for the AI intervention.	Page 6-8
	6b	Explanation for choice of comparators			Page 6-8
**Objectives**	7	Specific objectives or hypotheses			Page 8
**Trial design**	8	Description of trial design including type of trial (for example, parallel group, crossover, factorial, single group), allocation ratio, and framework (for example, superiority, equivalence, noninferiority, exploratory)			Page 8
Methods: participants, interventions and outcomes					
**Study setting**	9	Description of study settings (for example, community clinic, academic hospital) and list of countries where data will be collected. Reference to where list of study sites can be obtained	SPIRIT-AI 9 Extension	Describe the onsite and offsite requirements needed to integrate the AI intervention into the trial setting.	Page 9 & Appendix 2
**Eligibility criteria**	10	Inclusion and exclusion criteria for participants. If applicable, eligibility criteria for study centers and individuals who will perform the interventions (for example, surgeons, psychotherapists)	SPIRIT-AI 10 (i) Elaboration	State the inclusion and exclusion criteria at the level of participants.	Page 9-10 &
			SPIRIT-AI 10 (ii) Extension	State the inclusion and exclusion criteria at the level of the input data.	Page 11-15

Continued

Table 1 | SPIRIT-AI checklist (Continued)

Section	Item	SPIRIT 2013 item[a]	SPIRIT-AI item		Addressed on page number[b]
**Interventions**	11a	Interventions for each group with sufficient detail to allow replication, including how and when they will be administered	SPIRIT-AI 11a (i) Extension	State which version of the AI algorithm will be used.	Page 7-8&11-12
			SPIRIT-AI 11a (ii) Extension	Specify the procedure for acquiring and selecting the input data for the AI intervention.	Page 11-15
			SPIRIT-AI 11a (iii) Extension	Specify the procedure for assessing and handling poor-quality or unavailable input data.	Page 13-14
			SPIRIT-AI 11a (iv) Extension	Specify whether there is human–AI interaction in the handling of the input data, and what level of expertise is required for users.	Page 12
			SPIRIT-AI 11a (v) Extension	Specify the output of the AI intervention.	Page 11&15
			SPIRIT-AI 11a (vi) Extension	Explain the procedure for how the AI intervention's output will contribute to decision-making or other elements of clinical practice.	Page 12
	11b	Criteria for discontinuing or modifying allocated interventions for a given trial participant (for example, drug dose change in response to harms, participant request, or improving/worsening disease)			Page 19
	11c	Strategies to improve adherence to intervention protocols, and any procedures for monitoring adherence (for example, drug tablet return, laboratory tests)			Page 18
	11d	Relevant concomitant care and interventions that are permitted or prohibited during the trial			Page 10
**Outcomes**	12	Primary, secondary, and other outcomes, including the specific measurement variable (for example, systolic blood pressure), analysis metric (for example, change from baseline, final value, time to event), method of aggregation (for example, median, proportion), and time point for each outcome. Explanation of the clinical relevance of chosen efficacy and harm outcomes is strongly recommended			Page 15-17& 20-21
**Participant timeline**	13	Time schedule of enrollment, interventions (including any run-ins and washouts), assessments, and visits for participants. A schematic diagram is highly recommended (Fig. 1)			Page 24& figure4&5
**Sample size**	14	Estimated number of participants needed to achieve study objectives and how it was determined, including clinical and statistical assumptions supporting any sample size calculations			Page 19-20
**Recruitment**	15	Strategies for achieving adequate participant enrollment to reach target sample size			Page 17
Methods: assignment of interventions (for controlled trials)					
**Sequence generation**	16a	Method of generating the allocation sequence (for example, computer-generated random numbers), and list of any factors for stratification. To reduce predictability of a random sequence, details of any planned restriction (for example, blocking) should be provided in a separate document that is unavailable to those who enroll participants or assign interventions			Page 10-11

Continued

Table 1 | SPIRIT-AI checklist (Continued)

Section	Item	SPIRIT 2013 item[a]	SPIRIT-AI item	Addressed on page number[b]
**Allocation concealment mechanism**	16b	Mechanism of implementing the allocation sequence (for example, central telephone; sequentially numbered, opaque, sealed envelopes), describing any steps to conceal the sequence until interventions are assigned		Page 11
**Implementation**	16c	Who will generate the allocation sequence, who will enroll participants, and who will assign participants to interventions		Page 9&10 &17-18
**Blinding (masking)**	17a	Who will be blinded after assignment to interventions (for example, trial participants, care providers, outcome assessors, data analysts), and how		Page 11
	17b	If blinded, circumstances under which unblinding is permissible, and procedure for revealing a participant's allocated intervention during the trial		Not applicable
Methods: data collection, management and analysis				
**Data collection methods**	18a	Plans for assessment and collection of outcome, baseline, and other trial data, including any related processes to promote data quality (for example, duplicate measurements, training of assessors) and a description of study instruments (for example, questionnaires, laboratory tests) along with their reliability and validity, if known. Reference to where data collection forms can be found, if not in the protocol		Page 16-17 &18-19
	18b	Plans to promote participant retention and complete follow-up, including list of any outcome data to be collected for participants who discontinue or deviate from intervention protocols		Page 9-10&18
**Data management**	19	Plans for data entry, coding, security, and storage, including any related processes to promote data quality (for example, double data entry; range checks for data values). Reference to where details of data management procedures can be found, if not in the protocol		Page 18
**Statistical methods**	20a	Statistical methods for analyzing primary and secondary outcomes. Reference to where other details of the statistical analysis plan can be found, if not in the protocol		Page 20-21
	20b	Methods for any additional analyses (for example, subgroup and adjusted analyses)		Page 20-21
	20c	Definition of analysis population relating to protocol non-adherence (for example, as randomized analysis), and any statistical methods to handle missing data (for example, multiple imputation)		Page 20
Methods: monitoring				
**Data monitoring**	21a	Composition of data monitoring committee (DMC); summary of its role and reporting structure; statement of whether it is independnt from the sponsor and competing interests; and reference to where further details about its charter can be found, if not in the protocol. Alternatively, an explanation of why a DMC is not needed		Page 19
	21b	Description of any interim analyses and stopping guidelines, including who will have access to these interim results and make the final decision to terminate the trial		Page 19

Continued

Table 1 | SPIRIT-AI checklist (Continued)

Section	Item	SPIRIT 2013 item[a]	SPIRIT-AI item		Addressed on page number[b]
**Harms**	22	Plans for collecting, assessing, reporting, and managing solicited and spontaneously reported adverse events and other unintended effects of trial interventions or trial conduct	SPIRIT-AI 22 Extension	Specify any plans to identify and analyze performance errors. If there are no plans for this, justify why not.	Page 19
**Auditing**	23	Frequency and procedures for auditing trial conduct, if any, and whether the process will be independent from investigators and the sponsor			Page 18
Ethics and dissemination					
**Research ethics approval**	24	Plans for seeking research ethics committee/ institutional review board (REC/IRB) approval			Page 8&25
**Protocol amendments**	25	Plans for communicating important protocol modifications (for example, changes to eligibility criteria, outcomes, analyses) to relevant parties (for example, investigators, REC/IRBs, trial participants, trial registries, journals, regulators)			Page 25
**Consent or ascent**	26a	Who will obtain informed consent or assent from potential trial participants or authorized surrogates, and how (see Item 32)			Page 17&25
	26b	Additional consent provisions for collection and use of participant data and biological specimens in ancillary studies, if applicable			Not applicable
**Confidentiality**	27	How personal information about potential and enrolled participants will be collected, shared, and maintained in order to protect confidentiality before, during, and after the trial			Page 19&26
**Declaration of interests**	28	Financial and other competing interests for principal investigators for the overall trial and each study site			Page 26-27
**Access to data**	29	Statement of who will have access to the final trial dataset, and disclosure of contractual agreements that limit such access for investigators	SPIRIT-AI 29 Extension	State whether and how the AI intervention and/or its code can be accessed, including any restrictions to access or re-use.	Page 19
**Ancillary and post-trial care**	30	Provisions, if any, for ancillary and post-trial care, and for compensation to those who suffer harm from trial participation			Page 19&25-26
**Dissemination policy**	31a	Plans for investigators and sponsor to communicate trial results to participants, healthcare professionals, the public, and other relevant groups (for example, via publication, reporting in results databases, or other data sharing arrangements), including any publication restrictions			Page 26
	31b	Authorship eligibility guidelines and any intended use of professional writers			Page 26
	31c	Plans, if any, for granting public access to the full protocol, participant-level dataset, and statistical code			Page 26
Appendices					
**Informed consent materials**	32	Model consent form and other related documentation given to participants and authorized surrogates			Appendix 3
**Biological specimens**	33	Plans for collection, laboratory evaluation, and storage of biological specimens for genetic or molecular analysis in the current trial and for future use in ancillary studies, if applicable			Not applicable

[a]It is strongly recommended that this checklist be read in conjunction with the SPIRIT 2013 Explanation & Elaboration for important clarification on the items. [b]Indicates page numbers to be completed by authors during protocol development.
