# Supplementary material for: Effect of an artificial intelligence-assisted tool on non-valvular atrial fibrillation anticoagulation management in primary care: protocol for a cluster randomized controlled trial
Source: Trials. 2022 Apr 15;23:316. doi: 10.1186/s13063-022-06250-8 (PMC9013112; doi:10.1186/s13063-022-06250-8)
Supplement: Supplementary file 2 — Additional file 2. Community health centers participating in the study. [file 13063_2022_6250_MOESM2_ESM.pdf]

Appendix 1

Community health centers participating in the study

1. Caojiadu community health center
2. Changjiang Road community health center
3. Dachang community health center
4. Gaojing community health center
5. Jiang’an Temple community health center
6. Jiangning Road community health center
7. Juquan community health center
8. Second Shimen Road community health center
9. Sitang community health center
10. Songnan community health center
11. West Nanjing Road community health center
12. West Tianmu Road community health center
13. Wusong community health center
14. Youyi community health center
