# Supplementary material for: Effect of an artificial intelligence-assisted tool on non-valvular atrial fibrillation anticoagulation management in primary care: protocol for a cluster randomized controlled trial
Source: Trials. 2022 Apr 15;23:316. doi: 10.1186/s13063-022-06250-8 (PMC9013112; doi:10.1186/s13063-022-06250-8)
Supplement: Supplementary file 3 — Additional file 3. ICD-10 codes for AF and atrial flutter. [file 13063_2022_6250_MOESM3_ESM.pdf]

Appendix 2

ICD-10 codes

1. I48.200：chronic atrial fibrillation;
2. I48.100：persistent atrial fibrillation;
3. I48.900x015：first diagnosed atrial fibrillation;
4. I48.100x002：permanent atrial fibrillation;
5. I48.000：paroxysmal atrial flutter;
6. I48.100x003: long-standing persistent arial fibrillation;
7. I48.900x004：atrial fibrillation;
8. I48.900：atrial fibrillation or atrial flutter;
9. I48.900x003：atrial flutter;
10. I48.300：typical arial flutter;
11. I48.301：type I atrial flutter;
12. I48.400: untypical arial flutter;
13. I48.401：type II atrial flutter;
