# Supplementary material for: Effect of an artificial intelligence-assisted tool on non-valvular atrial fibrillation anticoagulation management in primary care: protocol for a cluster randomized controlled trial
Source: Trials. 2022 Apr 15;23:316. doi: 10.1186/s13063-022-06250-8 (PMC9013112; doi:10.1186/s13063-022-06250-8)
Supplement: Supplementary file 4 — Additional file 4. Informed consent. [file 13063_2022_6250_MOESM4_ESM.pdf]

知情同意书

受试者须知页

方案名称：非瓣膜性房颤社区 AI 辅助管理工具对提高社区房颤抗凝规范性的应用效果研究
主要研究者：潘志刚
申办者:复旦大学附属中山医院。

尊敬的受试者：

您被邀请参加非瓣膜性房颤社区 AI 辅助管理工具对提高社区房颤抗凝规范性的应用效果研究，该项研究由复旦大学附属中山医院提供支持。请仔细阅读本知情同意书并慎重做出是否参加本项研究的决定。参加这项研究完全是您自主的选择。作为受试者，您必须在加入临床研究前给出您的书面同意书。当您的研究医生或者研究人员和您讨论知情同意书的时候，您可以让他/她给您解释您看不明白的地方。我们鼓励您在做出参与此项研究的决定之前，和您的家人及朋友进行充分讨论。您有权拒绝参加本研究，也可随时退出研究，且不会受到处罚，也不会失去您应有的权利。若您正在参加别的研究，请告知您的研究医生或者研究人员。本研究的背景、目的、研究过程及其他重要信息如下：

一、研究背景

心房颤动（房颤）是临床最常见的心律失常之一,其患病率随年龄增加而增长。<60 岁者房颤患病率约为 1%,75~84 岁者患病率上升至 12%,80 岁以后可超过 1/3。其中大多数是非瓣膜性房颤。房颤发展过程中,脑卒中是最严重的并发症。房颤引起的脑卒中致残率、致死率均较高,给个人及社会带来巨大的社会经济负担。研究显示，规范的抗凝管理，即服用抗凝药物，如华法林和新型口服抗凝药（如利伐沙班、达比加群酯等），可以明显降低房颤患者卒中的发生率。但抗凝药物带来的出血等不良反应后果也很严重，尤其是老年人。因此，我国专家建议在启动抗凝之前首先需要做卒中风险及出血风险进行评估，针对老年患者还需要进行综合评估。但这项工作繁琐费时，经常被医生遗漏。

人工智能(artificial intelligence, AI)能够汇总整理海量医学数据和临床医学知识，具有精准分群和强化学习等技术优势。房颤相关的 AI 工具在发达国家得到广泛应用，帮助全科医生更好开展房颤诊治工作，既提升医生的工作效率，又可以降低错诊率提高用药规范率。本研究的 AI 辅助管理工具为复旦大学附属中山医院与平安医疗科技有限公司合作研发，安装在社区医生的工作电脑中，收集房颤患者相关的实验室检查结果和既往病史等信息，依照我国最新的房颤诊疗指南，计算患者的卒中风险、出血风险以及其他跌倒和心理状态方面的综合评估，为社区医生提供患者个体化的房颤诊疗决策建议，以期提高我国社区全科医生的房颤诊治能力和规范性，改善患者的健康结局。

二、研究目的

初步调查在上海的社区使用 CDSS 带来的房颤规范抗凝率变化及社区医生对本软件的接受程度，并收集一些合理的改进建议，为正式研究的顺利开展做准备。

三、研究过程

1. 多少人将参与这项研究？

大约(84)人将参与在(2)个不同的社区卫生服务中心开展的本项研究，大约(30-50)人会在本院参与本研究。

2. 研究步骤

如果您同意参加本研究，请您签署这份知情同意书。

在您入选研究前，医生将根据您的病史进行筛选检查。确定您可以参加本研究后，您将依据就诊的社区卫生服务中心不同进行分组，如果您就诊的社区卫生服务中心在本研究的干预组（即接诊您的全科医生的电脑上安装了本研究的辅助工具），医生为您诊治时将参考本辅助工具提供的建议，否则，医生将按照自己的临床经验和知识储备为您提供诊治。第一次就诊时，医生会为您提供房颤综合诊疗方案，包括需完善的辅助检查、综合评估结果、抗凝治疗方案及复诊时间等。之后您可以根据医生的治疗建议结合自身情况进行复诊。复诊时医生会询问您近期的出凝血血检查指标（INR）（如果您在口服华法林），近期有无发生卒中或出血，抗凝药服用剂量，及自我评估的任何不适情况。在开展指尖血 INR 检测的社区卫生服务中心，您可以采集指尖血进行 INR 检测。未开展此项检测的社区卫生服务中心需要您至上级医院检测。INR 是房颤口服华法林患者必须定期检查的指标，根据检查结果医生可以评估您的华法林用量是否合适，并及时调整。

3. 这项研究会持续多久？

研究自您同意参加并进行第一次就诊开始持续一年时间。期间我们会定期对您进行问卷随访，随访将持续至您第一次就诊后的第 12 个月。

您可以在任何时间选择退出研究而不会失去您本应获得的任何利益。然而，如果在研究途中您决定退出本研究，我们鼓励您先和您的医生商议。如果您出现严重的不良事件，或者您的研究医生觉得继续参加研究不符合您的最佳利益，他/她会决定让您退出研究。申办者或者监管机构也可能在研究期间终止研究。但您的退出不会影响您的正常医疗待遇与权益不受影响。

如果您因为任何原因从研究中退出，您可能被询问有关您参加研究的情况。如果医生认为需要，您也可能被要求进行实验室检查和体格检查。

4. 研究中收集的信息和生物标本

本研究不收集您的任何生物标本。就诊信息和数据保存在您所在区的区卫健委网络信息安全中心。用于研究的数据由专人进行匿名提取。就诊信息依照相关医疗安全法规进行保存和销毁。

四、风险与受益

1. 参加本研究的风险是什么？

参加本研究可能给您带来的风险如下。您应该和您的研究医生，或者您愿意，与您平日照看您的医生讨论一下这些风险。

研究期间，您可能会发生一些、所有或者不发生这些不良事件（病人或临床试验受试者接受一种药品/医疗器械等试验产品后出现的不良医学事件）、风险、不适、不方便，如：

1）存在信息安全方面的风险，但此方面风险极低。我们会尽全力保护您提供的信息不被泄露，在法律允许的范围内，尽一切努力保护您个人医疗资料的隐私。

2）开展指尖血 INR 检测的社区卫生服务中心，我们将严格按照血液标本采集规范对您操作，可能会有一些非常小的风险，包括短暂的疼痛、局部青紫，少数人会有轻度头晕，或极为罕见的针头感染。若发生与研究相关损害，我们的医疗团队会为您提供及时的救治。

如果在研究期间您出现任何不适，或病情发生新的变化，或任何意外情况，不管是否与研究有关，均应及时通知您的医生，他/她将对此作出判断并给与适当的医疗处理。

您在研究期间需要按时到医院随访，这将会占用您的一些时间，也可能给您造成麻烦或带来不方便。

2. 参加研究有什么受益？

直接受益：如果您同意参加本研究，您将不需往返上级医院，省去长时间排队候诊的时间，即可能获得规范化的房颤抗凝治疗方案，预防卒中、栓塞等房颤并发症的发生。

潜在受益：通过研究，我们将持续改善社区全科医生的房颤综合诊治水平，提高上海的基层医疗水平。我们希望从您参与的本研究中得到的信息在将来能够使您或与您病情相同的病人获益。同时还可根据研究中发现的问题继续改进现有软件，使其更好的服务于基层医疗单位。

五、备选的治疗方案

除了参与本研究，您可以接受您的家庭医生或上级医院医生提供的常规治疗。常规治疗即医生根据自己的临床经验和知识水平对您进行诊疗。

请您和您的医生讨论后决定是否参加本研究。

六、研究结果的使用和个人信息的保密

研究过程中，我们将收集您的病史信息、实验室检查结果和随访信息，为保证隐私我们将对您的部分信息进行编码，您的个人标识符（例如姓名、出生日期、地址、医保卡号等）将被代码（唯一的患者编号）代替，以便任何人无法确定您的身份。所有数据保存在主要研究者手中，保存地点在复旦大学附属中山医院，保存时间 10 年，后予以销毁。除本次研究外，将来不会再次利用到您的这些信息

在您和其他受试者的理解和协助下，通过本项目研究的结果可能会在医学杂志

上发表，但是我们会按照法律的要求为您的研究记录保密。研究受试者的个人信息将受到严格保密，除非应相关法律要求，您个人信息不会被泄露。必要时，政府管理部门和医院伦理委员会及其它相关研究人员可以按规定查阅您的资料。

七、关于研究费用及相关补偿

1. 研究所用的器械及相关检查费用

全科医生应用本研究的辅助管理工具对您进行综合诊疗评估并给出诊疗建议是免费的。在社区卫生服务中心或上级医院进行的任何方式的 INR 检测，均不在免费范围之内。对于您同时合并的其他疾病所需的常规治疗和检查，也不在免费的范围之内。

2. 参加研究的补偿

参加本研究的房颤患者没有额外的人民币的补偿。

3. 发生损害后的补偿

如果发生与该项研究相关的损伤，如发生个人数据泄漏或因指尖血 INR 检测时血液采集带来损害，您可获得由申办方（复旦大学附属中山医院潘志刚研究团队）为您提供的必要的免费治疗或根据中国有关法律进行的补偿。本研究的主要场所为社区卫生服务中心的全科诊室，社区卫生服务中心均具备一定的急救措施及设备。如有必要，立即由接诊的全科医生护送患者至最近的上级医院做进一步抢救。全科医生将治疗措施及如出现相关不良反应的处理措施以口头及书面形式告知患者，并确认患者已知晓理解。遇到高龄或存在一定认知障碍的患者，同时告知其家属做好监督护理工作。

八、受试者的权利和相关注意事项

1. 您的权利

在参加研究的整个过程中，您都是自愿的。如果您决定不参加本研究，也不会影响您应该得到的其他治疗。如果您决定参加，会要求您在这份书面知情同意书上签字。您有权在试验的任何阶段随时退出试验而不会遭到歧视或受到不公平的待遇，您相应医疗待遇与权益不受影响。

2. 注意事项

作为受试者，您需要提供有关自身病史和当前身体状况的真实情况；告诉研究医生自己在本次研究期间所发现的任何不适；告诉研究医生自己最近是否参与其他研究，或目前正参与其他研究。

九、试验的资金来源和可能的利益冲突

本研究的所有资金来源于主要研究者的课题经费。无潜在的利益冲突。

十、获知信息的相关联系方式

如果在研究过程中有任何重要的新信息，可能影响您继续参加研究的意愿时，您的医生将会及时通知您。如果您对自己的研究数据，或研究结束后您希望知道本研究的发现。您可以在任何时间提出有关本项研究的任何问题，并得到相应的解答，

请通过电话 13924272715 与茹雪颖联系。

伦理委员会已经审查通过该研究，如果您有与自身权利/权益相关的任何问题，或者您想反映参与本研究过程中遭遇的困难、不满和忧虑，或者想提供与本研究有关的意见和建议，请联系复旦大学附属中山医院伦理委员会，联系电话：021-31587871，电子邮件：ec@zs-hospital.sh.cn。

受试者签字页

知情同意声明：

我已被告知此项研究的目的、背景、过程、风险及获益等情况。我有足够的时间和机会进行提问，问题的答复我很满意。

我也被告知，当我有问题、想反映困难、顾虑、对研究的建议，或想进一步获得信息，或为研究提供帮助时，应当与谁联系。

我已经阅读这份知情同意书，并且同意参加本研究。

我知道我可以选择不参加此项研究，或在研究期间的任何时候无需任何理由退出本研究。

我已知道如果我的状况更差了，或者我出现严重的不良事件，或者我的研究医生觉得继续参加研究不符合我的最佳利益，他/她会决定让我退出研究。无需征得我的同意，资助方或者监管机构也可能在研究期间终止研究。如果发生该情况，医生将及时通知我，研究医生也会与我讨论我的其他选择。

我将得到这份知情同意书的副本，上面包含我和研究者的签名。

受试者签名：______________________　　　　日期：____________

（注：如果受试者无行为能力/限制行为能力时，则需法定代理人签名和签署日期）

法定代理人签字：_________________　　　　日期：____________

（注：如果受试者不能阅读该知情同意书时，则需一名独立见证人证明研究者已将知情同意书的所有内容告知了受试者，独立见证人需签名和签署日期）

独立见证人签字：_________________　　　　日期：____________

研究者签名：______________________　　　　日期：____________
