# Supplementary material for: Effect of an artificial intelligence-assisted tool on non-valvular atrial fibrillation anticoagulation management in primary care: protocol for a cluster randomized controlled trial
Source: Trials. 2022 Apr 15;23:316. doi: 10.1186/s13063-022-06250-8 (PMC9013112; doi:10.1186/s13063-022-06250-8)
Supplement: Supplementary file 5 — Additional file 5. Ethical approval document. [file 13063_2022_6250_MOESM5_ESM.pdf]

伦理委员会批准函

Ethics Committee Approval Letter

审查编号 Approval No.: B2021-579R

项目名称 Study Title	非瓣膜性房颤社区 AI 辅助管理工具研发及推广效果研究		
试验产品名称 Study Product Name	非瓣膜性房颤社区 AI 辅助管理工具	产品类别/规格 Product Category	NA
批准文号及发文单位(Approval No. and Issued By) NA		研究分期(Phase of Study) NA	
主要研究者(Principal Investigator) 潘志刚		申办者(Sponsor) 复旦大学附属中山医院	
审查方式及日期 (Type & Date of Review)	■会议审查 (Meeting Review) 2021 年 08 月 26 日<br>■快速审查(Expedited Review) 2021 年 09 月 14 日		
会议地点 (Meeting Location)	复旦大学附属中山医院 5 号楼 211 会议室		
会议出席情况 (Meeting Attendance)	出席(Attendance) 12 人，投票(Vote) 12 人，回避(Avoidance) 0 人		

下列研究相关文件已经批准

The following documents have been approved

见附件

审查决定 Decision for this proposal and have been [√]:

[√] 同意 Approval

持续审查频率：□3 个月/3 Months □6 个月/6 Months ■1 年/1Year □不适用/NA

批准函有效期至： 2022 年 09 月 14 日

主任委员/副主任委员签名 Chair/Vice Chair Signature: [signature]

批准日期 Approval Date: 2021.9.14

复旦大学附属中山医院伦理委员会(盖章)Stamp of ZSEC

复旦大学附属中山医院医学伦理委员会

A-024 版本：3.4

联系方式：上海市徐汇区枫林路 180 号 邮编：200032 电话：021-31587871 传真：021-31587851

扫描全能王 创建

伦理委员会批准函

Ethics Committee Approval Letter

审查编号 Approval No.: B2021-579R

附件

序号	文件名称	版本号/日期
1.	研究方案	V1.1,2021-07-28
2.	知情同意书	V1.2,2021-09-01
3.	研究者简历	NA
4.	研究人员名单	NA
5.	患者满意度调查问卷简易版本（PSQ-18）	NA
6.	Morisky 用药依从性问卷（MMAS-8）	NA
7.	社区全科医生对非瓣膜性房颤患者抗凝治疗的知-信-行调查问卷	NA
8.	社区医生培训内容大纲	NA
9.	病例报告表	V1.0,2021-07-11
10.	等级测评结论	NA
11.	房颤临床辅助决策系统产品技术要求	NA
12.	房颤临床辅助决策系统测试报告	V1.0
13.	器械供应方资质	NA
14.	受试者鉴认代码表	NA

复旦大学附属中山医院伦理委员会(盖章)Stamp of ZSEC

A-024 版本：3.4

联系方式：上海市徐汇区枫林路 180 号 邮编：200032 电话：021-31587871 传真：021-31587851

扫描全能王 创建

伦理委员会委员名单及出席情况

Ethics Committee Composition and Attendant

会议编号： 2021 年第 08 次会议　　　　审查日期：Date of Review： 2021 年 08 月 26 日

委员姓名和职称 Member Name and Title		从事专业 Profession	性别 Gender	工作单位 Working Place	出席者签名 Signature of Attending
姓名	职称/职务				
樊嘉** Fan Jia	教授 Professor	肝外科 Liver Surgery	男 Male	中山医院 Zhong Shan Hospital	
秦新裕* Qin Xinyu	教授 Professor	普外科 General Surgery	男 Male	中山医院 Zhong Shan Hospital	
高鑫* Gao Xin	教授 Professor	内分泌 Endocrinology	女 Female	中山医院 Zhong Shan Hospital	[illegible]
衡慧珠 Heng Huizhu	委员 Member	社会学 Sociology	女 Female	枫林街道 Fenglin Community	[illegible]
李雪宁 Li Xuening	主任药师 Chief Pharmacist	临床药理 Clinical Pharmacology	女 Female	中山医院 Zhong Shan Hospital	[illegible]
张博恒 Zhang Boheng	主任医师 Chief Physician	肝肿瘤内科 Hepatic Oncology	男 Male	中山医院 Zhong Shan Hospital	
孙元 Sun Yuan	委员 Member	律师 Lawyer	男 Male	上海管博律师事务所 Shanghai Guanbo Law Firm	[illegible]
李华茵 Li Huayin	主任医师 Chief Physician	呼吸科 Respiratory Medicine	女 Female	中山医院 Zhong Shan Hospital	[illegible]
柏瑾 Bai Jin	副主任医师 Vice Chief Physician	心内科 Cardiology	女 Female	中山医院 Zhong Shan Hospital	[illegible]
刘天舒 Liu Tianshu	主任医师 Chief Physician	肿瘤内科 Medical Oncology	女 Female	中山医院 Zhong Shan Hospital	[illegible]
姜红 Jiang Hong	教授 Professor	心内科 Cardiology	女 Female	中山医院 Zhong Shan Hospital	[illegible]
孙湛 Sun Zhan	主任医师 Chief Physician	急诊 Emergency	男 Male	中山医院 Zhong Shan Hospital	[illegible]
吴伟忠 Wu Weizhong	研究员 Professor	肝癌机制研究 Mechanism of Liver Cancer	男 Male	中山医院 Zhong Shan Hospital	[illegible]
俞梅蓉 Yu Meirong	高级会计师 Senior Accountant	医院管理 Hospital Management	女 Female	中山医院 Zhong Shan Hospital	[illegible]
杨梦婕# Yang Mengjie	助理研究员 Research Associate	伦理 Ethics Review	女 Female	中山医院 Zhong Shan Hospital	[illegible]

注：** 主任委员， * 副主任委员，# 委员/秘书

扫描全能王 创建

伦理委员会批准函

Ethics Committee Approval Letter

审查编号 Approval No.: **B2021-579(2)**

**项目名称** Study Title	非瓣膜性房颤社区 AI 辅助管理工具研发及推广效果研究		
**试验产品名称** Study Product Name	非瓣膜性房颤社区 AI 辅助管理工具	**产品类别/规格** Product Category	PA100
**批准文号及发文单位**(Approval No. and Issued By)<br>NA		**研究分期**(Phase of Study)<br>NA	
**主要研究者**(Principal Investigator)<br>潘志刚		**申办者**(Sponsor)<br>复旦大学附属中山医院	
**审查方式及日期** (Type & Date of Review)	□会议审查 (Meeting Review)<br>■快速审查(Expedited Review) 2021 年 11 月 09 日		
**会议地点** (Meeting Location)	NA		
**会议出席情况** (Meeting Attendance)	NA		

下列研究相关文件已经批准

The following documents have been approved

1. 研究方案:V1.2,2021-10-15
2. 知情同意书: V1.3,2021-10-27

审查决定 Decision for this proposal and have been [√]:

[√] 同意 Approval

持续审查频率： □3 个月/3 Months □6 个月/6 Months □1 年/1Year ■不适用/NA

主任委员/副主任委员签名 Chair/Vice Chair Signature: [illegible]

批准日期 Approval Date: 2021.11.11

复旦大学附属中山医院伦理委员会(盖章)Stamp of ZSEC

A-024 版本：3.4

联系方式：上海市徐汇区枫林路 180 号 邮编：200032 电话：021-31587871 传真：021-31587851

扫描全能王 创建

声明(Statement):
(请仔细阅读)

1. 复旦大学附属中山医院伦理委员会（以下简称本伦理委员会）的职责、人员组成、操作规范和记录遵循 ICH-GCP 及中华人民共和国国家药品监督管理局颁布的《药物临床试验质量管理规范》、《医疗器械临床试验质量管理规范》和《药物临床试验伦理审查工作指导原则》，并遵守中国相关法律和法规的规定。
2. **研究实施前提**：所有研究需经伦理委员会审查获得批准函后方可实施，实施过程应遵循伦理委员会批准的方案执行，应符合赫尔辛基宣言和 GCP 的基本原则。**特殊情况：** ⑴属**《人类遗传资源采集、收集、买卖、出口、出境审批行政许可事项》**规定范畴的研究，获得中国人类遗传资源管理工作办公室批准/备案后，将批准/备案成功结果书面递交伦理委员会备案后方可实施。⑵属**《需进行临床试验审批的第三类医疗器械目录》**内医疗器械的临床试验，获得国家药品监督管理局备案成功结果书面提交伦理委员会备案后方可实施。⑶属**需在国家药品监督管理局备案/默示许可的项目**，获得备案成功结果/默示许可的结果及时提交伦理委员会备案后方可实施。
3. 研究过程中，对研究方案和知情同意书等相关文件所作的任何修订，均需得到本伦理委员会审查同意后方可实施。
4. 在复旦大学附属中山医院发生的严重不良事件或可疑且非预期严重不良反应等安全性信息需按伦理委员要求及时递交，伦理委员会有权对其评估做出新的决定。
5. 方案违背和偏离需及时报告本伦理委员会。
6. 需按照伦理初始审查伦理批准函的持续审查频率和首次批准时间提交持续审查申请，确保批准函到期前 1 个月递交持续审查申请，以获得伦理委员会的批准。(本伦理委员会有权根据实际开展情况改变持续审查频率)
7. 暂停/提前终止临床试验，需及时书面向本伦理委员会提出申请。
8. 研究结束时，需及时向本伦理委员会递交结题报告及相关附件。
9. 本批准函可能在其他参加单位伦理委员会备案，如对审查结果有不同意见，请及时与本伦理委员会联系。

联系方式：上海市徐汇区枫林路 180 号　　邮编：200032　　电话：021-31587871　　传真：021-31587851

扫描全能王 创建

伦理委员会委员名单

Ethics Committee Composition

委员姓名和职称 Member Name and Title		从事专业 Profession	性别 Gender	工作单位 Working Place
姓名	职称/职务			
樊嘉** Fan Jia	教授 Professor	肝外科 Liver Surgery	男 Male	中山医院 Zhong Shan Hospital
秦新裕* Qin Xinyu	教授 Professor	普外科 General Surgery	男 Male	中山医院 Zhong Shan Hospital
高鑫* Gao xin	教授 Professor	内分泌 Endocrinology	女 Female	中山医院 Zhong Shan Hospital
衡慧珠 Heng Huizhu	委员 Member	社会学 Sociology	女 Female	枫林街道 Fenglin Community
李雪宁 Li Xuening	主任药师 Chief Pharmacist	临床药理 Clinical Pharmacology	女 Female	中山医院 Zhong Shan Hospital
张博恒 Zhang Boheng	主任医师 Chief Physician	肝肿瘤内科 Hepatic Oncology	男 Male	中山医院 Zhong Shan Hospital
孙元 Sun Yuan	委员 Member	律师 Lawyer	男 Male	上海管博律师事务所 Shanghai Guanbo Law Firm
李华茵 Li Huayin	主任医师 Chief Physician	呼吸科 Respiratory Medicine	女 Female	中山医院 Zhong Shan Hospital
柏瑾 Bai Jin	副主任医师 Vice Chief Physician	心内科 Cardiology	女 Female	中山医院 Zhong Shan Hospital
刘天舒 Liu Tianshu	主任医师 Chief Physician	肿瘤内科 Medical Oncology	女 Female	中山医院 Zhong Shan Hospital
姜红 Jiang Hong	教授 Professor	心内科 Cardiology	女 Female	中山医院 Zhong Shan Hospital
孙湛 Sun Zhan	副主任医师 Vice Chief Physician	急诊 Emergency	男 Male	中山医院 Zhong Shan Hospital
吴伟忠 Wu Weizhong	研究员 Professor	肝癌机制研究 Mechanism of Liver Cancer	男 Male	中山医院 Zhong Shan Hospital
俞梅蓉 Yu Meirong	高级会计师 Senior Accountant	医院管理 Hospital Management	女 Female	中山医院 Zhong Shan Hospital
杨梦婕# Yang Mengjie	助理研究员 Research Associate	伦理 Ethics Review	女 Female	中山医院 Zhong Shan Hospital

注：** 主任委员， * 副主任委员，# 委员/秘书

扫描全能王 创建
