# Supplementary material for: Effect of an artificial intelligence-assisted tool on non-valvular atrial fibrillation anticoagulation management in primary care: protocol for a cluster randomized controlled trial
Source: Trials. 2022 Apr 15;23:316. doi: 10.1186/s13063-022-06250-8 (PMC9013112; doi:10.1186/s13063-022-06250-8)
Supplement: Supplementary file 6 — Additional file 6. Copy of the original funding documentation. [file 13063_2022_6250_MOESM6_ESM.pdf]

上海市卫生健康委
先进适宜技术推广项目

Shanghai Municipal Health Commission
the Advanced and Appropriate Technology Promotion Project

计划任务书

Planning assignment

Project ID
项目编号： 2019SY009

Project title Development and promotion of an AI-assisted tool for NVAF management in primary care
项目名称： 非瓣膜性房颤社区AI辅助管理工具研发及推广效果研究

Project undertaking unit (seal) Zhongshan Hospital, Fudan University
项目承担单位（盖章）： 复旦大学附属中山医院

Director Zhigang Pan
项目负责人： 潘志刚

联合单位（盖章）：

Time limit From July 2019 to June 2022
项目年限： 2019 年 7 月 至 2022 年 6 月

Application Date April 30th 2019
填报日期： 2019 年 4 月 30 日

Telephone
联系电话： 18616881251

Email
电子邮箱： zhigang_pan@163.com

上海市卫生健康委

二〇一九年

Shanghai Municipal Health Commission
2019

八、审核意见 Review Opinions

**项目责任单位意见** Opinons of the undertaking unit<br><br>本单位将按本任务书中所填报内容完成各项任务，并承诺保证所需人力、物力等各项保障。<br>We promised to complete the assignments in the application documents, and guarantee the necessary manpower and equipments.<br><br>法定代表人签字：<br>Signature of the legal representative<br>单位盖章：<br>Seal of the undertaking unit<br>二○一九年十月十二日<br>October 12nd, 2019
**上级主管部门意见** Opinions of the superior authority<br><br>同意上报 Approval<br><br>Seal 单位盖章：<br>二○一九年十月十二日<br>October 12nd, 2019
**上海市卫生健康委意见**<br>Opinions of Shanghai Municipal Health Commission<br><br>单位盖章：<br>Seal<br>二○　年　月　日
